# Gut microbial subtypes and clinicopathological value for colorectal cancer

**DOI:** 10.1002/cam4.70180

**Published:** 2024-09-05

**Authors:** Shuwen Han, Jing Zhuang, Yifei Song, Xinyue Wu, Xiaojian Yu, Ye Tao, Jian Chu, Zhanbo Qu, Yinhang Wu, Shugao Han, Xi Yang

**Affiliations:** ^1^ Huzhou Central Hospital, Affiliated Central Hospital Huzhou University Huzhou China; ^2^ Fifth Affiliated Clinical Medical College of Zhejiang Chinese Medical University, Huzhou Central Hospital Huzhou China; ^3^ Key Laboratory of Multiomics Research and Clinical Transformation of Digestive Cancer Huzhou China; ^4^ Institut Catholique de Lille, Junia (ICL), Université Catholique de Lille, Laboratoire Interdisciplinaire des Transitions de Lille (LITL) Lille France; ^5^ Shanghai Biozeron Biotechnology Co., Ltd. Shanghai China; ^6^ The Second Affiliated Hospital of Zhejiang University School of Medicine Hangzhou China

**Keywords:** clinicopathological features, colorectal cancer, gut bacteria, gut microbial subtypes, unsupervised clustering

## Abstract

**Background:**

Gut bacteria are related to colorectal cancer (CRC) and its clinicopathologic characteristics.

**Objective:**

To develop gut bacterial subtypes and explore potential microbial targets for CRC.

**Methods:**

Stool samples from 914 volunteers (376 CRCs, 363 advanced adenomas, and 175 normal controls) were included for 16S rRNA sequencing. Unsupervised learning was used to generate gut microbial subtypes. Gut bacterial community composition and clustering effects were plotted. Differences of gut bacterial abundance were analyzed. Then, the association of CRC‐associated bacteria with subtypes and the association of gut bacteria with clinical information were assessed. The CatBoost models based on gut differential bacteria were constructed to identify the diseases including CRC and advanced adenoma (AA).

**Results:**

Four gut microbial subtypes (A, B, C, D) were finally obtained via unsupervised learning. The characteristic bacteria of each subtype were *Escherichia‐Shigella* in subtype A, *Streptococcus* in subtype B, *Blautia* in subtype C, and *Bacteroides* in subtype D. Clinical information (e.g., free fatty acids and total cholesterol) and CRC pathological information (e.g., tumor depth) varied among gut microbial subtypes. *Bacilli*, *Lactobacillales*, etc., were positively correlated with subtype B. Positive correlation of *Blautia*, *Lachnospiraceae*, etc., with subtype C and negative correlation of *Coriobacteriia*, *Coriobacteriales*, etc., with subtype D were found. Finally, the predictive ability of CatBoost models for CRC identification was improved based on gut microbial subtypes.

**Conclusion:**

Gut microbial subtypes provide characteristic gut bacteria and are expected to contribute to the diagnosis of CRC.

## 1 INTRODUCTION

Colorectal cancer (CRC) is characterized by low 5‐year survival rate and high mortality rate, and a concerning shift toward younger age at diagnosis, contributing to a significant global disease burden.^1^, ^2^ CRC is prone to metastasis, mainly accounting for CRC‐related deaths.^3^ Early‐stage CRC can be effectively treated with surgery, but the insidious symptoms often lead to diagnoses at intermediate or advanced stages, causing patients to miss the optimal window for treatment.

Numerous studies have suggested the strong link between gut bacteria and CRC.^4^, ^5^, ^6^ As the main inhabitants of the gut, gut bacteria can be classified into beneficial bacteria (e.g., *Bifidobacterium* and *Clostridium butyricum*), harmful bacteria (e.g., *Escherichia coli* and *Staphylococcus aureus*), and CRC‐related bacteria based on their different roles in disease progression.^7^, ^8^, ^9^, ^10^ Differences in the abundance and community composition of gut bacteria across diverse populations create a foundation for considering distinctive gut bacteria as potential biomarkers for CRC.^11^, ^12^, ^13^ For instance, differential gut bacteria (e.g., *Bifidobacterium* and *norank*_*f_*_*Oscillospiraceae*, *Eisenbergiella*) are enriched in poorly differentiated CRC patients, which suggests a potential link between the degree of CRC pathological differentiation and gut bacteria.^11^ Furthermore, evidence supports the association between clinically collected information (e.g., age, obesity, alcohol abuse, and smoking) and CRC.^14^, ^15^, ^16^ Finally, gut bacteria are related to drug efficacy, and they show an important role in the treatment of CRC. Dysregulation in gut bacterial significantly reduced the efficacy of 5‐Fluorouracil in homologous transplanted CRC mice, resulting in increased tumor weight and volume. This highlights the vital role of gut bacteria in regulating the host's response to chemotherapeutic drugs.^17^

Currently, the molecular typing of CRC based on the heterogeneity of CRC molecular characteristics has made rapid progress and greatly promotes the development of individualized precision therapy.^18^, ^19^ For example, the consensus molecular subtype (CMS) based on gene expression is widely recognized, and it has important implications in the selection of highly effective drugs for CRC in preclinical models.^20^, ^21^, ^22^ In addition, the CMS classification is successfully applied to demonstrate the heterogeneity of colorectal adenomas. It was discovered that CMS3, the most prevalent type of adenoma (73%), has the lowest risk of progression to CRC.^23^ Nevertheless, the characteristic microbiota typing for CRC has not yet been explored.

In this study, CRC typing based on gut bacteria was conducted via unsupervised clustering, aiming to explore potential molecular targets for CRC stratification and diagnosis from the perspective of gut microbes. Moreover, the correlation between gut bacteria and clinical information was analyzed. Our findings are expected to provide potential biomarkers for the identification of CRC and point out a new direction for clinical diagnosis of the disease.

## 2 METHODS

### 2.1 Volunteer recruitment and data acquisition

A total of 914 volunteers were recruited from the Huzhou Central Hospital from January 1, 2020, to December 31, 2022, including 376 patients with CRC, 363 patients with advanced adenoma (AA), and 175 normal controls, and their stool samples were collected. Both CRC and AA patients included in this study were pathologically diagnosed. The exclusion criteria of CRC patients are as follows: (1) Other malignancies exist in the patient; (2) other serious cardiopulmonary diseases exist in patients; (3) a history of antibiotic, hormonal, or gut microbiota use in the 3 months prior to admission existed in patients; (4) other intestinal diseases including ulcerative colitis and Crohn's disease exist in patients; and (5) mental illness or cognitive impairment exists in patients.

The Huzhou Central Hospital ethics committee (no.: 20191101–01) and Chinese clinical trial registry (http://www.chictr.org.cn, no.: ChiCTR2100050167) approved the plan involving the patients' clinical and informed consent. Besides, the corresponding clinical information (e.g., age and gender) and pathological characteristics were collected from the hospital information management system (Table 1).

**TABLE 1:** Clinical information before gut typing.

	Group			*p*‐value
	CRC	AA	NC	
	(*n* = 376)	(*n* = 363)	(*n* = 175)	
Gender				
Male	218	220	108	0.653
Female	158	143	67	
Age	63 ± 16.873	59 ± 10.163	57 ± 22.871	0.071
Family history				
Yes	2	0	1	0.37
No	374	363	174	
Diabetes				
Yes	32	30	8	0.231
No	344	333	167	
Hypertension				
Yes	121	122	57	0.363
No	255	241	118	
Smoking				
Yes	131	119	55	0.502
No	245	244	120	
Drinking				
Yes	57	51	32	0.152
No	319	312	143	

### 2.2 Data processing and clustering

16S rRNA sequencing was used to detect gut bacterial sequences of the stool samples. The processing of microbial sequencing data can refer to previous studies.^11^

Unsupervised clustering was carried out by R software (Version:4.3.1).^24^ ConsensusClusterPlus package was employed to perform consensus clustering on gut bacterial metagenomics data. This clustering divided the samples into subtypes, facilitating subsequent comparative analyses among different subtypes. The specific parameters used included maxK = 9, reps = 50, pItem = 0.8, pFeature = 1, distance = “Euclidean,” and clusterAlg = “km.”^25^ The probably approximately correct (PAC) algorithm was used to select the best *K* value to optimize the clustering model.

### 2.3 Data Analysis

Descriptive analysis, difference analysis, correlation analysis, and CatBoost model construction were performed on gut bacteriological data among different diseases and subtypes.

The genus‐level data were visualized via the R and ggplot2 packages for species composition among different subtypes, and cluster composition in different diseases and disease composition in different clusters were also presented. Besides, principal coordinate (PCoA) analysis and non‐metric multidimensional scaling (NMDS) analysis for different diseases and subtypes were carried out, with specific steps referring to previous studies.^11^

SPSS 27.0 statistical software was used to analyze clinical information for differences among different subtypes, and linear discriminant analysis and the Kruskal–Wallis tests were used to screen for differential bacteria in different diseases and subtypes. Additionally, clinical information with statistical differences (*p* < 0.05) was further analyzed by the Kruskal–Wallis tests among gut microbial subtypes.

Spearman correlation coefficients were calculated for different diseases and subtypes to describe the association of CRC‐associated bacteria and gut microbial characteristics with diseases and subtypes.

Differential bacteria were included to construct CatBoost models for disease identification and the construction method can be referred to previous studies.^11^ During model construction, the data were divided into 80% of training set to build the model and 20% of test set to verify the model. Subtype B has no validation set due to small sample size.

The flow chart of this study is shown in Figure S1.

## 3 RESULTS

### 3.1 Gut microbial subtypes based on unsupervised learning algorithm

**FIGURE 1:**
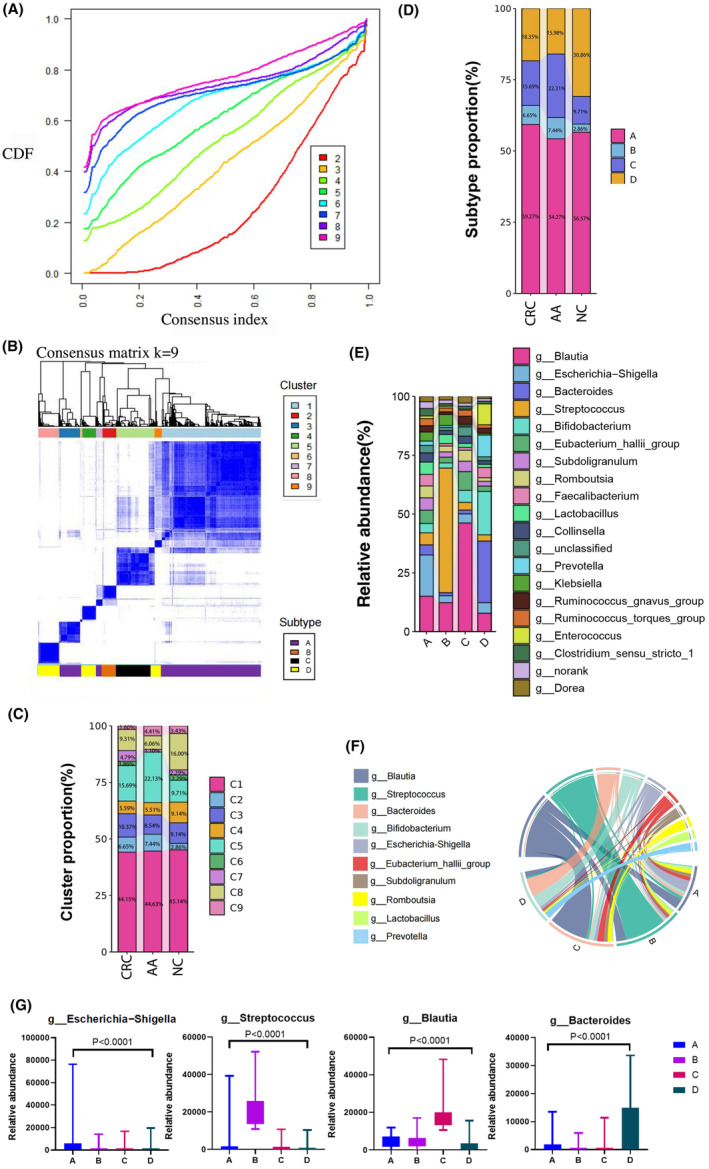
Gut microbial typing and their composition analysis. (A) Consensus cumulative distribution function (CDF). *K* represents the number of clusters, and different colors represent CDF curves with different *K* values. The higher the stability of CDF curve, the more reliable the clustering results corresponding to the *K* value. (B) Consensus matrix heat map. Cluster refers to the number of cluster subtypes, and subtype refers to the number of gut microbial subtypes. The clustering results for *K* = 9 were displayed. (C) Cluster composition of colorectal diseases. The proportion of 9 clusters in different colorectal disease populations including CRC, AA, NC was shown. (D) Subtype composition of colorectal diseases. The proportion of 4 gut microbial subtypes in different colorectal disease populations including CRC, AA, NC was shown. (E) Bacterial structure of gut microbial subtypes. The composition of the top 20 gut bacteria in the four gut microbial subtypes was shown. (F) Chord diagram of gut bacteria among different subtypes. Different colors correspond to gut microbial subtypes. The longer the string, the greater the correlation. (G) Characteristic gut bacteria of gut microbial subtypes. The abundance of *Escherichia‐Shigella*, *Streptococcus*, *Blautia*, *Bacteroides* in four gut microbial subtypes was analyzed.

The unsupervised training of gut bacteria from three populations, namely CRC, AA, and NC, showed that the best effect was achieved with a cluster number of 9 (Figure 1A). The ratio of people with disease status (AA and CRC) to NC varied across clusters (Table 2). C2 showed the highest ratio (CRC+AA/NC=10.4), followed by C5 (CRC+AA/NC=8.23). The ratios of C1 (CRC+AA/NC=4.15), C3(CRC+AA/NC=4.38), C4 (CRC+AA/NC=2.56), C6 (CRC+AA/NC=1.75), C7 (CRC+AA/NC=5.5), C8 (CRC+AA/NC=2.04), and C9 (CRC+AA/NC=3.67) were close. According to the ratio, nine clusters were classified into four subtypes (Figure 1B, Table 3). Subtype B showed the highest ratio (CRC+AA/NC=10.40), followed by subtype C (CRC+AA/NC=8.24), subtype A (CRC+AA/NC=4.24), and subtype D (CRC+AA/NC=2.35) (Table 4).

**TABLE 2:** Composition and ratio of clusters with a clustering number of 9.

Cluster	CRC	AA	NC	Sum	CRC+NC	CRC+AA	AA+NC	AA/NC	CRC/NC	CRC+AA/NC
C1	166	162	79	407	245	328	241	2.05	2.1	4.15
C2	25	27	5	57	30	52	32	5.4	5	10.4
C3	39	31	16	86	55	70	47	1.94	2.43	4.38
C4	21	20	16	57	37	41	36	1.25	1.31	2.56
C5	59	81	17	157	76	140	98	4.76	3.47	8.24
C6	7	0	4	11	11	7	4	0	1.75	1.75
C7	18	4	4	26	22	22	8	1	4.5	5.5
C8	35	22	28	85	63	57	50	0.79	1.25	2.04
C9	6	16	6	28	12	22	22	2.67	1	3.67
Sum	376	363	175	914						

**TABLE 3:** Cluster structure of subtypes.

Subtype	Cluster
A	C1, C3, C7
B	C2
C	C5
D	C4, C6, C8, C9

**TABLE 4:** Composition and ratio of subtypes.

Subtype	CRC	AA	NC	Sum	*X* ^2^	*p*	CRC+NC	AA+NC	CRC+AA	AA/NC	CRC/NC	CRC+AA/NC	*X* ^2^	*p*
A	223	197	99	519	362	<0.001	322	296	420	1.99	2.25	4.24	70	<0.001
B	25	27	5	57			30	32	52	5.40	5.00	10.40		
C	59	81	17	157			76	98	140	4.76	3.47	8.24		
D	69	58	54	181			123	112	127	1.07	1.28	2.35		
Sum	376	363	175	914										

The composition of clusters and subtypes varied in different populations (Figure 1C,D). C1, C5, and C3 were the top three clusters in CRC and AA, while C1, C8, and C5 were the top three clusters in NC. Subtype A and D were the top two subtypes in CRC and NC, while subtype A and C were the top two subtypes in AA. Significant structural differences in gut bacteria at the genus level were found among the four subtypes (Figure 1E). For example, *Escherichia‐Shigella*, *Blautia*, and *Eubacterium_hallii_group* were the top three bacteria in subtype A, while *Bacteroides*, *Bifidobacterium*, and *Prevotella* were the top three bacteria in subtype D. What's more, there were also differences in the correlation between gut bacteria and subtypes (Figure 1F). *Escherichia‐Shigella* showed a higher correlation with subtype A, while *Streptococcus* showed a higher correlation with subtype B. *Romboutsia*, *Blautia*, etc. were more strongly associated with subtype C, while *Bacteroides*, *Bifidobacterium*, etc. were more strongly associated with subtype D. Additionally, important characteristic gut bacteria of four subtypes were shown (Figure 1G). *Bacteroides* were enriched in subtype D, and *Blautia* showed higher abundance in subtype C. *Escherichia‐Shigella* were abundant in subtype A, and *Streptococcus* were enriched in subtype B.

Before subtype classification, the clustering effect was not satisfactory (Figure S2A,B). After subtypes were formed, the clustering effect was improved (Figure S2C–J). Subtype B had the best clustering effect (Figure S2D,H), followed by subtype D (Figure S2F,J).

### 3.2 Clinical information relationship with gut microbial subtypes

Clinically available information, such as gender and age, varied among the four subtypes (Table 5). In addition to the differences in the number of patients with CRC among the four subtypes, there were statistically significant differences in platelet, alanine aminotransferase, aspartate‐aminotransferase, free fatty acids, and total cholesterol (*p* < 0.05).

**TABLE 5:** Clinical information of different subtypes.

	A	B	C	D	*p*‐value
	(*n* = 519)	(*n* = 57)	(*n* = 157)	(*n* = 181)	
Number of CRCs					
/	223	25	59	69	<0.001
Gender					
Male	336	41	100	124	0.831
Female	183	16	57	57	
Age					
/	59 ± 18.719	58 ± 14.547	56 ± 10.401	53 ± 20.812	0.189
Diabetes					
Yes	34	5	17	14	0.357
No	405	52	140	167	
Hypertension					
Yes	195	29	55	59	0.201
No	324	28	102	112	
Smoking					
Yes	133	16	45	45	0.837
No	386	41	112	136	
Drinking					
Yes	93	10	32	33	0.914
No	426	47	125	148	
Fecal occult blood test					
Positive	276	32	80	83	0.312
Negative	139	12	43	57	
BMI					
/	23.494 ± 3.243	23.244 ± 3.917	22.926 ± 3.222	22.916 ± 3.353	0.184
Red blood cell					
/	4.464 ± 2.334	4.387 ± 0.527	4.384 ± 0.553	4.363 ± 0.510	0.932
Hemoglobin					
/	132.901 ± 17.833	133.204 ± 16.113	134.170 ± 17.515	130.378 ± 17.631	0.34
White blood cell					
/	5.625 ± 2.049	6.120 ± 2.689	5.879 ± 2.250	5.867 ± 2.469	0.338
Platelet					
/	194.130 ± 69.607	189.818 ± 63.146	187.829 ± 62.639	213.664 ± 93.118	0.018
Red blood cell distribution width value					
/	13.482 ± 2.150	13.386 ± 1.661	13.327 ± 2.042	13.508 ± 2.507	0.894
Alanine aminotransferase					
/	20.424 ± 14.873	29.783 ± 21.936	23.052 ± 16.385	19.265 ± 13.346	<0.001
Aspartate‐aminotransferase					
/	22.006 ± 7.992	27.852 ± 13.981	22.939 ± 11.934	23.335 ± 11.756	0.003
Total protein					
/	68.728 ± 9.575	66.711 ± 7.227	68.473 ± 6.548	67.353 ± 7.127	0.229
Albumin					
/	40.633 ± 14.987	39.470 ± 3.450	40.723 ± 3.379	39.967 ± 4.673	0.869
Total bilirubin					
/	13.948 ± 6.768	13.363 ± 6.029	13.791 ± 6.765	13.019 ± 6.279	0.534
Direct bilirubin					
/	5.320 ± 2.366	5.234 ± 2.172	5.071 ± 2.001	4.940 ± 2.168	0.33
Creatinine					
/	69.736 ± 15.195	73.604 ± 14.594	69.848 ± 13.061	66.792 ± 16.888	0.052
Urea nitrogen					
/	5.232 ± 3.448	5.373 ± 1.706	5.169 ± 1.335	4.831 ± 1.572	0.482
Glucose					
/	5.197 ± 2.332	4.782 ± 0.954	5.238 ± 1.746	5.314 ± 1.699	0.518
Triglyceride					
/	1.553 ± 1.246	1.801 ± 1.228	1.790 ± 1.730	1.426 ± 0.828	0.078
Total cholesterol					
/	4.600 ± 0.947	4.271 ± 0.956	4.760 ± 1.379	4.465 ± 0.891	0.022
High density lipoprotein					
/	47.387 ± 12.148	45.608 ± 11.758	44.455 ± 11.653	45.252 ± 12.181	0.063
Low density lipoprotein					
/	102.235 ± 32.842	90.242 ± 21.020	104.980 ± 32.508	101.115 ± 29.067	0.063
Lipoprotein A					
/	23.359 ± 25.553	19.211 ± 22.378	25.186 ± 25.327	22.392 ± 25.327	0.575
Apolipoprotein A					
/	3.363 ± 11.055	2.589 ± 8.183	3.140 ± 12.104	4.650 ± 0.979	0.582
Free fatty acids					
/	455.056 ± 218.846	447.661 ± 221.294	386.752 ± 210.601	429.295 ± 217.182	0.022

**FIGURE 2:**
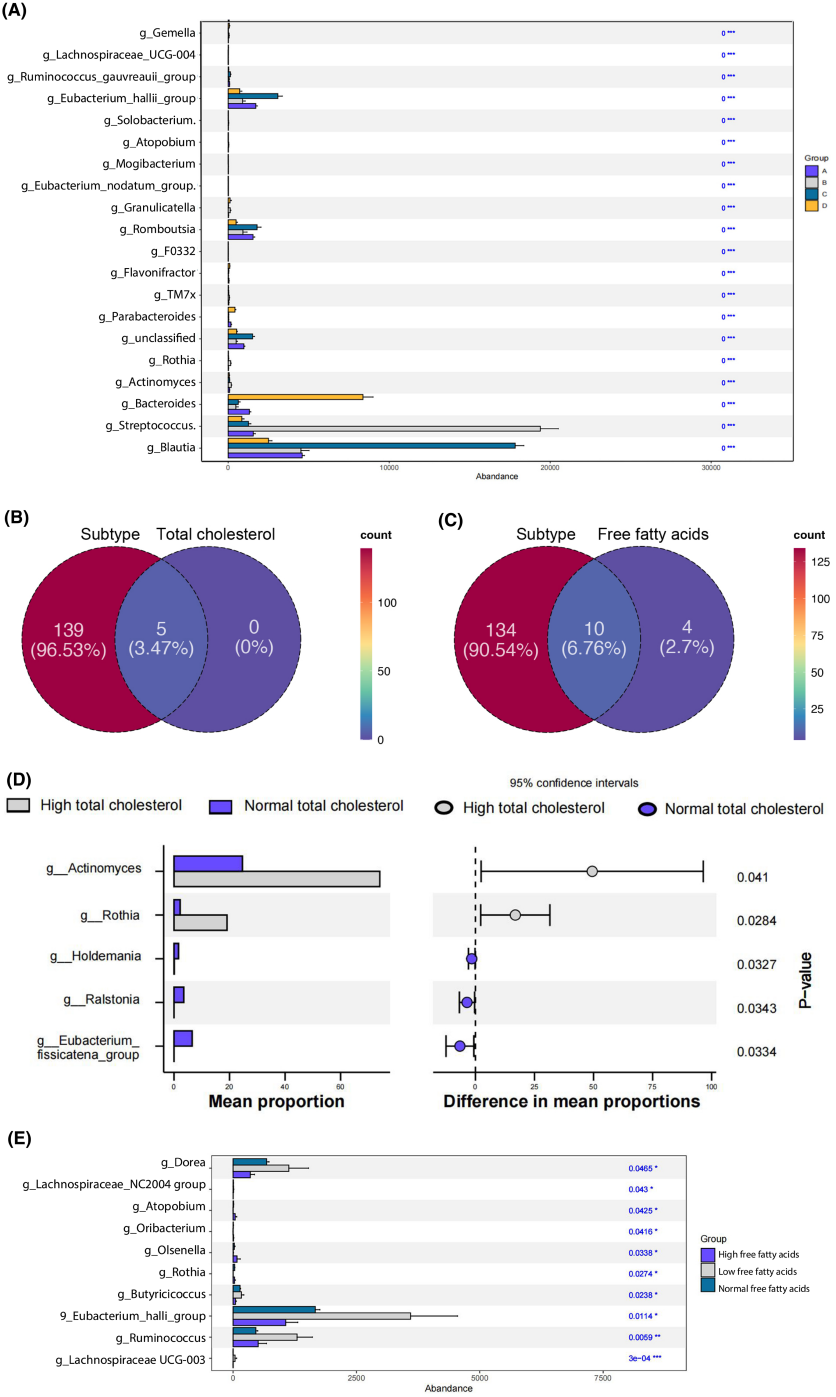
Differences in the abundance of the common gut bacteria in specific clinical information groups. (A) Differential bacteria among four subtypes. The top 20 differential bacteria among four gut microbial subtypes were shown. “***” means 0.0001 < *p* ≤ 0.001, with significant statistical difference. (B): Venn diagram for subtype and total cholesterol. The orange circle represents the differential bacteria among different gut microbial subtypes, the pink circle represents the differential bacteria between different total cholesterol levels, and the overlapping part of the circle represents the common differential bacteria. (C) Venn diagram for subtype and free fatty acid. The orange circle represents the differential bacteria among different gut microbial subtypes, the pink circle represents the differential bacteria among different free fatty acid levels, and the overlapping part of the circle represents the common differential bacteria. (D) Common differential bacteria in different subtypes and total cholesterol content. (E) Common differential bacteria in different subtypes and free fatty acid. “*” 0.01 < *p* ≤ 0.05, “**” 0.001< *p* ≤ 0.01, and “***” 0.0001 < *p* ≤ 0.001.

To better interpret the relationship between clinical information and gut microbial subtype, differential gut bacteria that played an important role in both gut microbial subtype and important clinical information were screened. The top 20 differential gut bacteria among different subtypes were displayed in Figure 2A. For example, *Streptococcus* showed higher abundance in subtype B, *Blautia* were enriched in subtype C, and *Bacteroides* were more abundant in subtype D.

In total, 5 (3.47%) gut bacteria were found to vary both in different subtypes and total cholesterol content (Figure 2B). *Actinomyces* and *Rothia* were significantly enriched in high total cholesterol group, while *Holdemania*, *Eubacterium fissicatena group*, etc. were enriched in normal total cholesterol group (Figure 2D). Ten (6.76%) common bacteria were found in different subtypes and free fatty acid content (Figure 2C). Furthermore, *Eubacterium_hallii_group*, *Ruminococcus*, *Dorea*, etc. showed higher abundance in low free fatty acids group (Figure 2E).

Moreover, the common differential gut bacteria for platelet, alanine aminotransferase, aspartate‐aminotransferase are shown in Figure S3.

### 3.3 CRC pathological characteristics relationship with gut microbial subtypes

Based on the high sensitivity of tumor markers in screening, the examination of tumor markers provides a reliable reference for clinical diagnosis. Differential expression of nine tumor markers, such as CA125 and CA153, among four subtypes was presented (Figure S4A). Notably, CA19‐9 was overexpressed in subtype B (*p* = 0.0089).

Pathological information, including degree of differentiation and tumor depth, of CRC patients in each gut microbial subtype was analyzed (Figure S4B–I). For example, adenocarcinoma (94.11%), well‐differentiated (68.18%), and lymph nodes without metastasis (68.18%) were relatively enriched in subtype B. The ulcerative type (54.55%) was the characteristic tumor shape of subtype D, and the tumor tended to occur in the colon (56.06%).

### 3.4 CRC‐associated bacteria among different gut microbial subtypes

**FIGURE 3:**
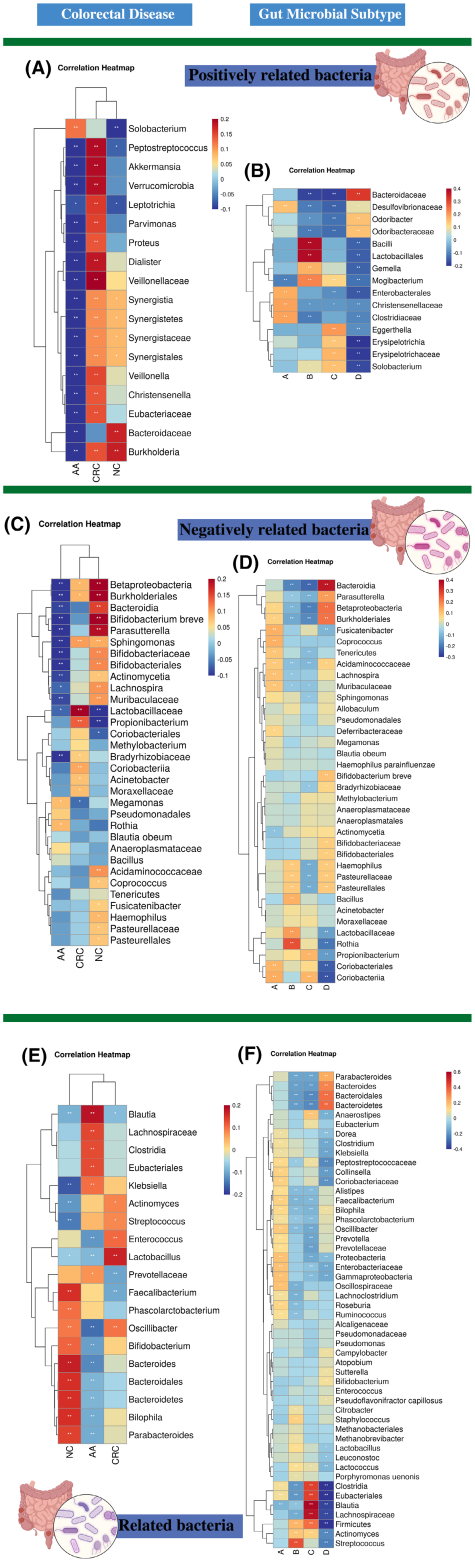
Correlation of CRC‐associated bacteria with different colorectal diseases and subtypes. (A) Correlation heat map of positively related bacteria for colorectal diseases. (B) Correlation heat map of positively related bacteria for gut microbial subtypes. (C) Correlation heat map of negatively related bacteria for colorectal diseases. (D) Correlation heat map of negatively related bacteria for gut microbial subtypes. (E) Correlation heat map of related bacteria for colorectal diseases. (F) Correlation heat map of related bacteria for gut microbial subtypes. Colorectal diseases include CRC, AA, NC, and gut microbial subtypes include subtype A, B, C, and D. The sign of the correlation coefficient *r* is independent of the magnitude of the correlation, with “+” representing a positive correlation and “‐” representing a negative correlation. The darker the color, the greater the correlation. “*” 0.01 < *p* ≤ 0.05, “**” 0.001 < *p* ≤ 0.01, “***” 0.0001< *p* ≤ 0.001.

This study identified CRC‐associated bacteria, beneficial bacteria, and harmful bacteria via the literature review. The association between characteristic gut bacteria and different diseases and subtypes is expected to provide insight into potential links between disease states and gut microbial subtypes. Bacteria with significant correlations with different populations were shown (|*r*| > 0.14) in Figure 3A. The significant negative correlation was found between positively related bacteria (e.g., *Burkholderia*, *Veillonellaceae*) and AA. *Bacilli* (*r* = 0.38) and *Lactobacillales* (*r* = 0.38) were positively correlated with subtype B, and *Solobacterium* (*r* = −0.18), *Erysipelotrichia* (*r* = −0.20), etc. were negatively correlated with subtype D (Figure 3B). In addition, it was found that *Fusobacterium*, a representative positively related bacterium of CRC, was found to be significantly enriched in subtypes A and D. For NC, AA, and CRC, there were differences in bacterial abundance among each subtype, but no statistical significance was discovered (Figure S5).

Negatively related bacteria, including *Betaproteobacteria* (*r* = 0.28) and *Burkholderiales* (*r* = 0.28), were significantly positively correlated with NC, while *Lactobacillaceae* (*r* = −0.16) and *Propionibacterium* (*r* = −0.11) were negatively correlated. *Burkholderiales* (*r* = −0.30), *Betaproteobacteria* (*r* = −0.30), and *Sphingomonas* (*r* = −0.18) were found to have a significant negative correlation with AA (Figure 3C). The significant positive correlation existed between *Bacteroidia* and subtype D (*r* = 0.36), and between *Rothia* and subtype B (*r* = 0.27). *Coriobacteriia* (*r* = −0.25), *Coriobacteriales* (*r* = −0.24), etc. were negatively correlated with subtype D (Figure 3D).

There was a significant positive correlation between CRC‐related bacteria (e.g., *Bacteroides* and *Faecalibacterium*) and NC. *Blautia* (*r* = 0.18) was positively correlated with AA, and *Oscillibacter* (*r* = −0.15) was negatively correlated with AA. The significant positive correlation was found between *Lactobacillus* and CRC (*r* = 0.17) (Figure 3E). *Blautia* (*r* = 0.59), *Lachnospiraceae* (*r* = 0.56), *Clostridia* (*r* = 0.45), *Eubacteriales* (*r* = 0.45), etc. were found to be positively correlated with subtype C. Moreover, *Blautia* (r = −0.36), *Clostridia* (*r* = −0.40), *Eubacteriales* (*r* = −0.40), *Firmicutes* (*r* = −0.43), etc. were also found to be negatively correlated with subtype D (Figure 3F). In addition, intra‐group correlations of beneficial and harmful bacteria were analyzed for different diseases and subtypes (Figure S6).

### 3.5 Gut microbial characteristics among different microbial subtypes

**FIGURE 4:**
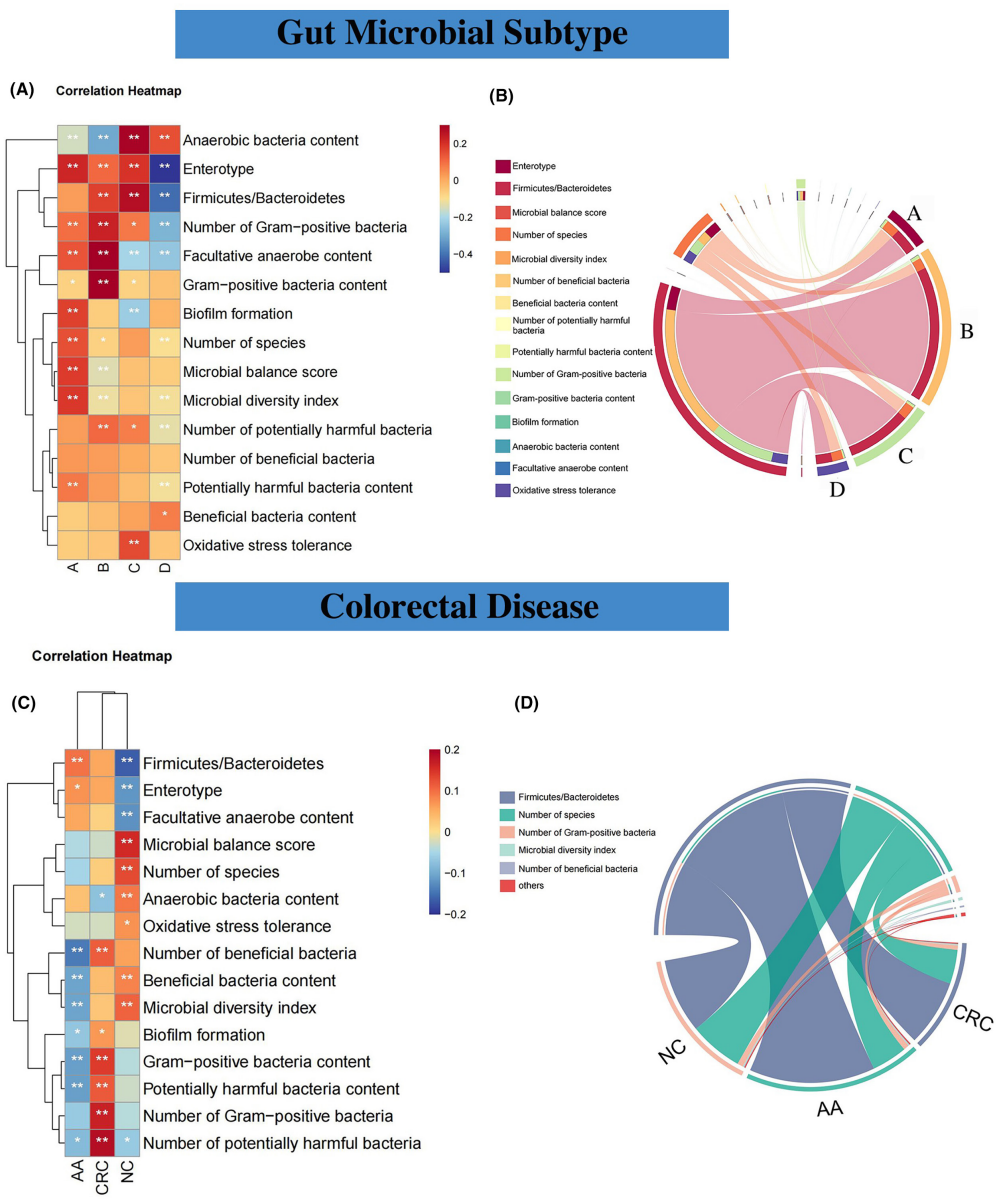
Correlation of gut microbial characteristics with diseases and subtypes and CRC prediction effect of CatBoost models. (A) Correlation heat map of gut microbial characteristics for gut microbial subtypes. (B) Chord diagram of gut microbial characteristics among different subtypes. (C) Correlation heat map of gut microbial characteristics for colorectal diseases. (D) Chord diagram of gut microbial characteristics among different diseases. Prediction targets of CatBoost models include CRC, AA, and CRC/AA, with different ranges of colorectal disease populations for the three targets. The accuracy, sensitivity, and specificity of the model are the comparison indexes before and after classification.

As the main inhabitants of the gut, gut bacteria have a crucial impact on gut health and the overall situation of gut bacteria can be described by the following 15 characteristics. Considering the potential association between the gut microbial characteristic and gut bacterial subtype, the intra‐group correlation among different subtypes and various characteristics was analyzed (Figure 4A).

#### 3.5.1 Number of species

The number of strains detected above the minimum threshold (at least one test sequence) revealed a notable positive correlation with subtype A, with a correlation coefficient of 0.15 in our results.

#### 3.5.2 Enterotype

According to the species and abundance of human intestinal flora, the intestinal microecology can be classified into three enterotypes, namely, *Prevotella*, *Bacteroides*, and *Ruminococcus*.^26^ Different degrees of positive correlation existed between enterotype and subtype A (*r* = 0.22), B (*r* = 0.11), and C (*r* = 0.20), while there was negative correlation with subtype D (*r* = −0.55).

#### 3.5.3 *Firmicutes*/*Bacteroidetes*

The *Firmicutes*/*Bacteroidetes* ratio is an indicator of diseases, including obesity and inflammatory bowel disease.^28^ The ratio was positively correlated with subtype B (*r* = 0.17) and C (*r* = 0.27), while D was negatively correlated with the ratio (*r* = −0.40).

#### 3.5.4 Anaerobic bacteria content

Intestinal flora can be divided into predominant microflora and sub‐dominant microflora according to their number. Most of the predominant microflora are obligatory anaerobic bacteria and play an important guiding role in the physiological and pathological functions of the whole flora. Subtype A (*r* = −0.15) and B (*r* = −0.30) were negatively correlated with anaerobic bacteria content, while subtype C (*r* = 0.29) and D (*r* = 0.15) were positively correlated.

#### 3.5.5 Facultative anaerobe content

The sub‐dominant microflora are mainly aerobic bacteria or facultative anaerobic bacteria and have potential pathogenicity. Contrary to the results of anaerobic bacteria content, subtype C (*r* = −0.21) and D (*r* = −0.23) were negatively correlated with facultative anaerobe content, while subtype A (*r* = 0.14) and B (*r* = 0.31) were positively correlated.

#### 3.5.6 Number of beneficial bacteria

Gut bacteria are divided into beneficial and harmful bacteria based on physiological function. Subtype A, B, and C were positively correlated with number of beneficial bacteria, but there was no statistical significance (*p* > 0.05).

#### 3.5.7 Beneficial bacteria content

The dominance of beneficial bacteria inhibits the propagation of pathogenic bacteria to help maintain intestinal health, while the dominance of harmful bacteria may lead to diseases, such as diarrhea and CRC. Subtype D was found to have a positive correlation with beneficial bacteria content (*r* = 0.08).

#### 3.5.8 Number of potentially harmful bacteria

In a healthy gut, beneficial and harmful bacteria are in a dynamic balance. Under the induction of environmental factors, potentially harmful bacteria may break the balance and induce disease. The number of potentially harmful bacteria was positively correlated with subtype B (*r* = 0.11) and C (*r* = 0.08), while it was negatively correlated with subtype D (*r* = −0.13).

#### 3.5.9 Potentially harmful bacteria content

The increase in potentially harmful bacteria could fuel the increase in disease risk. Subtype A was positively correlated with the potentially harmful bacteria content (*r* = 0.09).

#### 3.5.10 Microbial balance score

The balance of microecology ensures the normal physiological functions of the host, including digestion and immunity. Subtype A was significantly positively correlated with the microbial balance score (*r* = 0.18).

#### 3.5.11 Microbial diversity index

A well‐balanced and highly diverse gut flora contributes to a healthy gut. Subtype A was significantly positively correlated with the microbial diversity index (*r* = 0.19).

#### 3.5.12 Number of gram‐positive bacteria

The results proved that the number of gram‐positive bacteria was significantly positively correlated with subtype B (*r* = 0.11), while it was negatively correlated with subtype D (*r* = −0.13).

#### 3.5.13 Gram‐positive bacteria content

Subtype B was found to be positively correlated with the gram‐positive bacteria content (*r* = 0.35).

#### 3.5.14 Oxidative stress tolerance

Subtype C was positively correlated with the oxidative stress tolerance (*r* = 0.16).

#### 3.5.15 Biofilm formation

Biofilms play a key role in gut diseases, such as providing a protective environment for pathogens that evade host immunity. The results revealed that biofilm formation was positively correlated with subtype A (*r* = 0.17), but negatively correlated with subtype C (*r* = −0.22).

To fully understand the relationship between the gut microbial characteristics and gut microbial subtype, the intergroup correlation among gut bacterial subtypes and microbial characteristics was analyzed (Figure 4B). For instance, *Firmicutes*/*Bacteroidetes* had a higher correlation with subtype B.

Additionally, considering the potential association between gut bacteria and different colorectal diseases, differential bacteria were included for intra‐group and intergroup correlation analysis for different diseases (Figure 4C,D). AA was negatively correlated with the number of beneficial bacteria (*r* = −0.14), potentially harmful bacteria content (*r* = −0.11), etc.; CRC was positively correlated with the number of potentially harmful bacteria (*r* = 0.18), number of gram‐positive bacteria (*r* = 0.16), etc.; NC was positively correlated with microbial balance score (*r* = −0.16) and negatively correlated with *Firmicutes*/*Bacteroidetes* (*r* = 0.16); *Firmicutes*/*Bacteroidetes* showed a higher correlation with AA, while the number of species, the number of gram‐positive bacteria and microbial diversity index, etc. had a higher correlation with NC.

### 3.6 Improved CRC identification performance based on gut microbial subtypes

The abundance difference in gut bacteria from 914 stool samples was analyzed by linear discriminant analysis and the Kruskal–Wallis test. For example, *Lactobacillaceae*, *Bacilli*, etc. were enriched in CRC, while *Clostridia*, *Firmicutes*, etc. were enriched in AA (Figure S7A). The abundance of *Bacteroides* was higher in NC, while the abundance of *Lactobacillus* was higher in CRC (Figure S7B). There were common differential bacteria both in diseases and subtypes (Figure S6). For instance, the same differential bacteria of subtype A, such as *Faecalibacterium* in NC, *Lactobacillaceae* in CRC, and *Erysipelatoclostridiaceae* in AA.

Based on the important differential bacteria screened by the Kruskal–Wallis test, CatBoost models were established according to different gut microbial subtypes for the identification of colorectal diseases, including AA and CRC. After the establishment of subtypes, the disease prediction ability of machine learning model was significantly improved. The sample size for subtype B was only 57 cases (25 CRC, 5 NC, and 27 AA), so there was no test set. The disease identification models of subtype C performed best, with the accuracy of 78.57% for CRC, 90.00% for AA, and 93.55% for CRC/AA (Figure S8, Table 6).

**TABLE 6:** Disease identification models.

	Normal control	Subtype			
		A	B	C	D
CRC					
*Discovery set*					
Accuracy (%)	77.05	78.21	100.00	96.77	90.00
Specificity (%)	75.58	76.75	100.00	100.00	96.00
Sensitivity (%)	88.24	89.66	100.00	96.00	84.00
Test set					
Accuracy (%)	70.27	72.31	N/A	78.57	82.61
Specificity (%)	73.91	72.88	N/A	50.00	90.91
Sensitivity (%)	100.00	66.67	N/A	90.00	75.00
AA					
*Discovery set*					
Accuracy (%)	83.02	83.97	100.00	94.87	96.63
Specificity (%)	82.58	81.25	100.00	100.00	95.74
Sensitivity (%)	84.54	95.56	100.00	94.12	97.62
Test set					
Accuracy (%)	75.00	79.66	N/A	90.00	69.57
Specificity (%)	73.96	77.55	N/A	100.00	77.78
Sensitivity (%)	83.33	90.00	N/A	89.47	64.29
CRC+AA					
*Discovery set*					
Accuracy (%)	81.78	84.82	98.25	95.24	87.59
Specificity (%)	81.60	84.21	98.11	100.00	86.21
Sensitivity (%)	100.00	100.00	100.00	94.92	93.10
Test set					
Accuracy (%)	80.43	82.69	N/A	93.55	75.00
Specificity (%)	80.87	82.35	N/A	100.00	78.57
Sensitivity (%)	100.00	100.00	N/A	93.33	62.50

## 4 DISCUSSION

In this study, unsupervised clustering was used for classification, and four gut microbial subtypes were finally obtained according to the proportion of CRC. Considering the close association between gut bacteria and CRC, the correlation among gut microbial subtypes, clinical information and CRC pathological characteristics was analyzed, and significant differences in the correlation among them were found. Moreover, the correlation between CRC‐associated bacteria and gut microbial characteristics explored in previous studies with different diseases and different subtypes was analyzed. Finally, the predictive ability of disease identification models based on different gut microbial subtypes was significantly optimized, which showed the potential of gut microbial subtypes for the diagnosis and prevention of CRC.

Based on the dominant bacterial species in the human gut, the concept of “enterotype” proposed in 2011 has the same classification potential as blood type, which is currently a hot research topic.^26^ Inspired by that, gut microbial subtypes were proposed based on unsupervised clustering and disease (CRC+AA) proportion in this study for the first time. Unsupervised learning has been successfully practiced in analyzing disease subphenotypes and their corresponding clinical information, aiming at stratified diagnosis and treatment.^29^ In this study, the human gut microbiota were classified into nine clusters via unsupervised clustering. Among the nine gut subtypes, C2 showed the highest CRC+AA/NC ratio, followed by C5. Meanwhile, C2 and C5 were subtypes B and C, which were at high risk of CRC. The cluster with high disease proportion was more consistent with the gut microbiota profile of CRC.

According to the disease (CRC+AA) proportion, the clusters with relatively close disease proportions in the remaining clusters were merged, and human gut microbiota were finally divided into four subtypes. Gut microbial subtypes were not related to age, gender, and other factors (*p* > 0.05), which showed the similar stability as the “enterotype.”^26^ The classification of the gut based on the distinctive features of gut bacteria derived from CRC has the potential to enhance population‐specific, stratified diagnosis and treatment, thus offering valuable clinical guidance for the identification of CRC. Besides, the characteristic bacteria of each subtype were found, namely *Bacteroides* of subtype D, *Blautia* of subtype C, *Escherichia‐Shigella* of subtype A, and *Streptococcus* of subtype B, respectively. Recently, the association between *Bacteroides* and CRC was strongly supported by the discovery through Mendelian randomization that 13 unclassified *Bacteroides* genera increased the risk of CRC by 2%–15%.^30^ Besides, *Escherichia‐Shigella* that could promote inflammatory bowel disease (IBD) was highly enriched in subtype A.^31^ The “driver‐passenger” model of CRC indicated that IBD was an important predisposition factor for CRC mediated by “driver” bacteria.^32^ Therefore, it is necessary to pay attention to the relationship among IBD, gut bacteria, and CRC, which could provide directions for the diagnosis and prevention of CRC for subtype A. The correlation among gut microbial subtypes and gut microbial characteristics was analyzed, which indicated the interaction between gut microbiota and hosts. The expansion of facultative anaerobes and the decrease in obligate anaerobes can disrupt gut microbiota homeostasis and ultimately accelerate the occurrence and progression of chronic inflammation and CRC.^33^ In this study, the content of anaerobic bacteria was negatively correlated with subtype B, while facultative anaerobic bacteria contents were positively correlated with subtype B. For subtype B, with the highest proportion of CRC, the changed contents of facultative and anaerobic bacteria provide directions for the diagnosis and treatment of CRC. Subtype C was significantly positively correlated with *Lachnospiraceae* family, including *Blautia*, which is an important producer of short‐chain fatty acids in the gut.^34^ Although a few members of the *Lachnospiraceae* family were harmful to the host, such as the pro‐inflammatory effects of *Blautia gnavus*, a recent study revealed that *Lachnospiraceae* family bacteria inhibited the progression of CRC by promoting the immune monitoring function of CD8^+^T cells collectively.^35^, ^36^

Different subtypes of gut microbiota had different risks of developing CRC. The criteria to measure CRC risk could be complex, including the proportion of the disease population in each subtype, and the correlation with CRC‐associated bacteria and gut microbial characteristics. First, the proportion of CRC and AA of gut microbial subtypes has a significant impact on the risk of CRC. Gut microbiota of CRC and AA have remarkable changes, which provides the promising biomarkers for CRC diagnosis.^37^, ^38^ The accumulation of CRC and precancerous lesions AA patients makes the gut microbial characteristics of gut subtypes more similar to those of CRC. Moreover, the correlation between CRC‐associated bacteria and each subtype reflects the CRC risk of gut subtypes from the perspective of gut microbes to some extent. Subtype that has a high positive correlation with positively related bacteria, a high negative correlation with negatively related bacteria, and a high correlation with related bacteria will be considered as a high CRC risk gut microbial subtype. For instance, *Bacilli* and *Lactobacillales*, which were identified as positive related bacteria for CRC, and it was found that they showed a significant positive correlation with subtype B, which indicated an increased risk of CRC for subtype B.^39^, ^40^ *Erysipelotrichia*, *Erysipelotrichaceae*, *Solobacterium*, etc., have been identified as positive related bacteria for CRC in the previous study, and it was found that they had a significant negative correlation with subtype D in this study, which suggested a relatively low CRC risk of subtype D.^40^, ^41^, ^42^ Additionally, the association of gut microbial characteristics with different subtypes, such as the contents of facultative anaerobes and obligate anaerobes associated with the imbalance of gut microecology, can be an important reference to measure the risk of CRC in different subtypes. In general, subtype B could rank first for the risk of CRC, followed by subtype C, subtype A, and subtype D according to our results.

It was found that lipid metabolism is associated with gut microbial subtypes. High levels of cholesterol that are associated with westernized diet are regarded as a potential risk of increased CRC.^39^ Many patients with CRC have abnormal lipid metabolism, and this was associated with altered gut microbiota in previous studies, which provided an opportunity for cholesterol accumulation that may further exacerbate CRC progression.^43^, ^44^ In this study, statistical differences of total cholesterol were found among the four gut microbial subtypes, and the highest content was found in subtype C whose CRC/NC ratio was relatively high. The results indicated that cholesterol may be related to the classification of gut types, thus making the gut microbial subtypes more convincing to a certain extent. Further research is required to focus on the links among cholesterol, CRC, and gut bacteria. It was found that *Actinomyces* have cholesterol‐degrading capabilities and are highly enriched in moderately differentiated CRC patients with the potential as a biological target for the degree of CRC differentiation.^11^, ^45^ Function prediction and analysis of *Actinomyces* illustrated that it can induce high expression of TLR2, TLR4, and NF‐κB in young‐onset CRC and reduce infiltration of CD8^+^T cells in tumor microenvironment, which could provide a direction for the mechanism study of CRC induced by *Actinomyces*.^46^ There is substantial evidence that gut bacteria are involved in cholesterol metabolism, and abnormal cholesterol metabolism is a hallmark of CRC.^44^, ^47^, ^48^ In this study, *Actinomycetes* were enriched in the high cholesterol group at the genus level, but the clinical genotypes of *Actinomyces* were diverse, which suggested the necessity of exploring the important role of *Actinomyces* in cholesterol metabolism at a more precise taxonomic level in the future.^49^

However, there are still some limitations in this study. First, as the largest number of inhabitants in the gut, enteroviruses were not included in the sequencing. In the future, enterovirus should be included in research to explore the interaction between enterovirus and bacteria in CRC, which could provide a comprehensive interpretation perspective of the involvement of gut microbes in the occurrence and development of CRC. Subsequently, the mechanism of characteristic differential gut bacteria in the progression of CRC needs to be further supplemented with animal experiments. Most healthy volunteers are not willing to take the initiative to undergo colonoscopy, so the number of NC in this study is relatively small. Therefore, in the future, the sample size will be further expanded, especially NC. Moreover, there is a lack of validation cohorts to demonstrate the reliability of gut microbial subtypes. Therefore, more people from different geographies need to be included to verify the suitability of gut microbial subtypes. By doing so, the gut microbial subtype is expected to be further popularized, which could accelerate its progress into clinical practice for the diagnosis of CRC.

## 5 CONCLUSION

Gut microbial subtypes are not affected by most clinical information (such as age and gender) and are associated with the pathological characteristics of CRC. The correlation of CRC‐associated bacteria among four subtypes was different, which reflects the bacteriological characteristics of each subtype. In addition, the differences in the correlation of gut microbial characteristics among four subtypes provide a direction for future research on the mechanism of microbe‐host interaction in CRC. Most importantly, the gut microbial subtype via unsupervised clustering provides diagnostic biomarkers for CRC stratification.

## AUTHOR CONTRIBUTIONS

**Shuwen Han:** Writing – original draft (equal). **Jing Zhuang:** Data curation (equal); formal analysis (equal); writing – original draft (equal). **Yifei Song:** Formal analysis (equal). **Wu Xinyue:** Formal analysis (equal). **Yu Xiaojian:** Formal analysis (equal). **Tao Ye:** Methodology (equal); validation (equal). **Jian Chu:** Data curation (equal); supervision (equal). **Zhanbo Qu:** Data curation (equal); supervision (equal); visualization (equal). **Yinhang Wu:** Visualization (equal). **Shugao Han:** Conceptualization (equal); methodology (equal). **Xi Yang:** Conceptualization (equal); methodology (equal); writing – original draft (equal).

## FUNDING INFORMATION

This work was supported by the Key research and development project of Science and Technology Department of Zhejiang Province (no.: 2022C03026), the Public Welfare Technology Application Research Program of Huzhou (no.: 2023GZ86), the Zhejiang Medical and Health Technology Project (no.: 2023RC274), and China University Industry University Research Innovation Fund (no.: 2023HT078).

## CONFLICT OF INTEREST STATEMENT

The authors declare that no potential conflicts of interest exist.

## ETHICS STATEMENT

The clinical protocols involving the patients and the informed consent form were approved by the Chinese Clinical Trial Registry (http://www.chictr.org.cn, ChiCTR2100050167) and Ethics Committee of Huzhou Central Hospital (20191101–01). All participants provided written informed consent. All methods were performed in accordance with the relevant guidelines and regulations in ethics approval and consent to participate.

## Supporting information


Figure S1.

Figure S2.

Figure S3.

Figure S4.

Figure S5.

Figure S6.

Figure S7.

Figure S8.

## Data Availability

The datasets generated during the current study are not publicly available but obtained from corresponding authors on reasonable request.
