# Supplementary figures and images for: Gut microbial subtypes and clinicopathological value for colorectal cancer
Source: Cancer Med. 2024 Sep 5;13(17):e70180. doi: 10.1002/cam4.70180 (PMC11375334; doi:10.1002/cam4.70180)

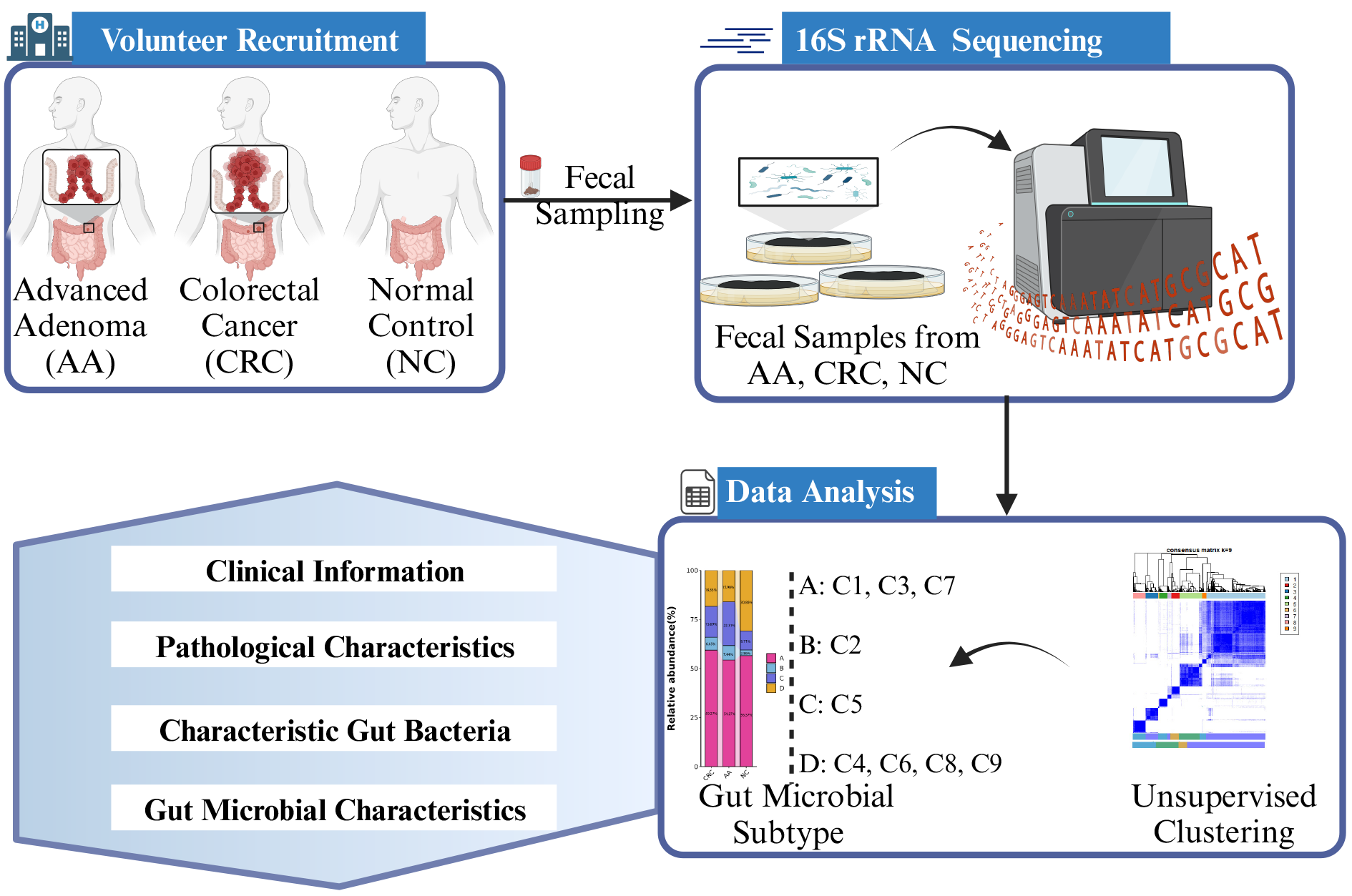

Supplement: Supplementary file 1 — Figure S1. [file CAM4-13-e70180-s008.tif]

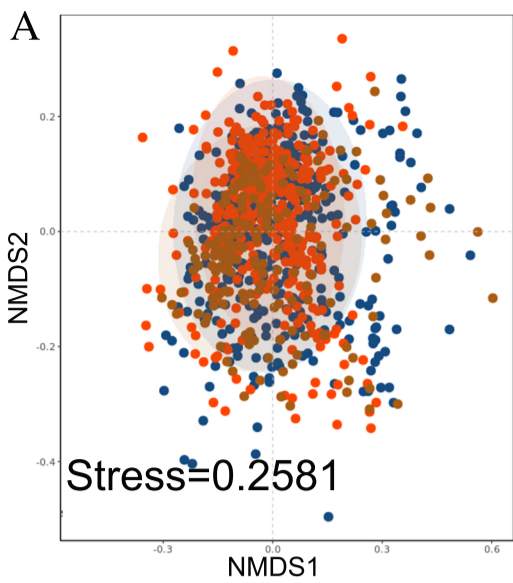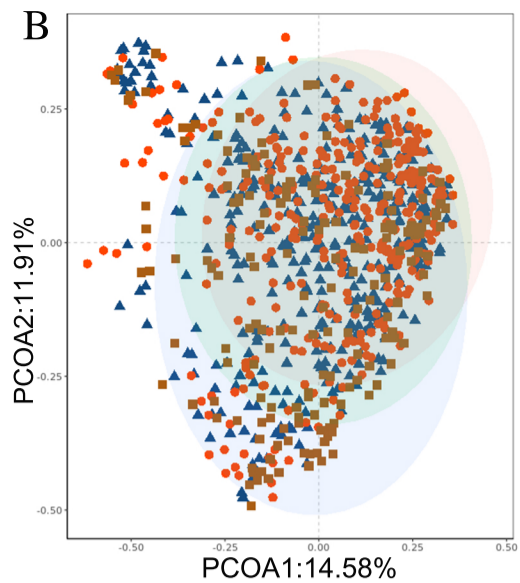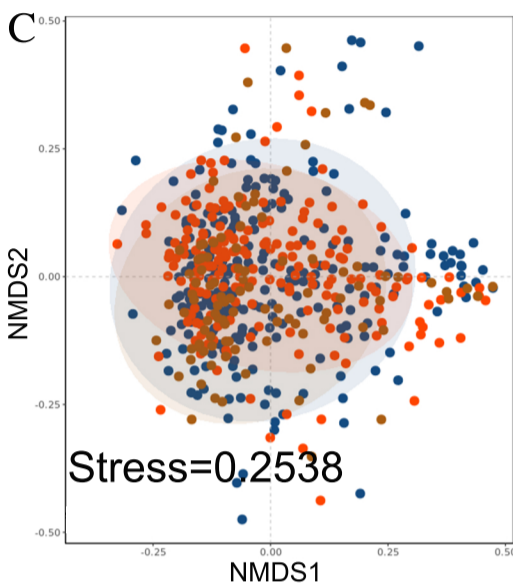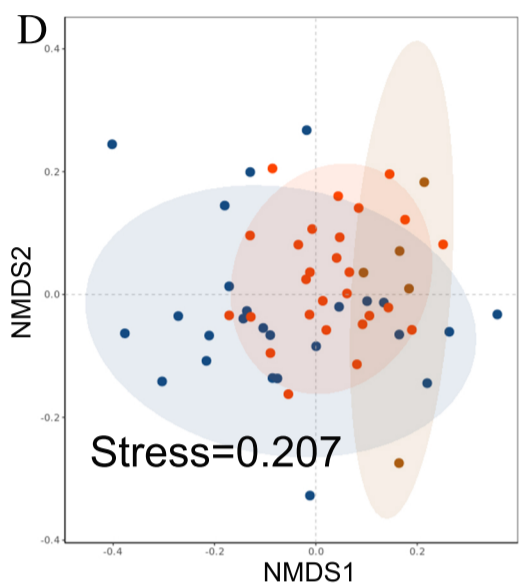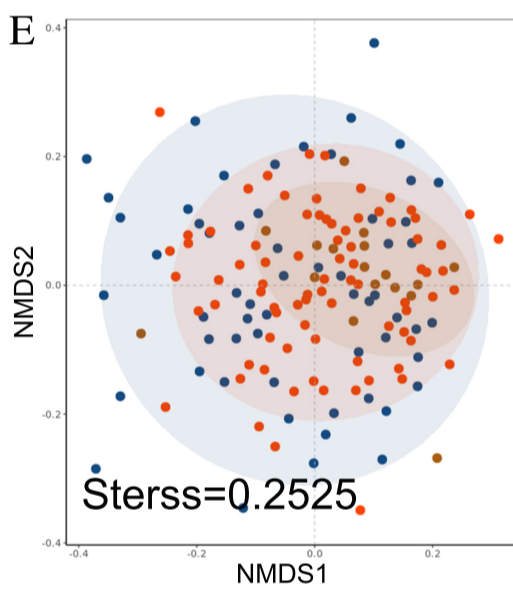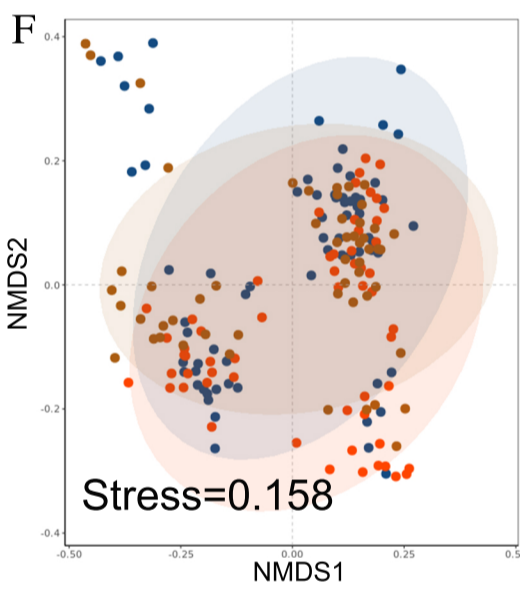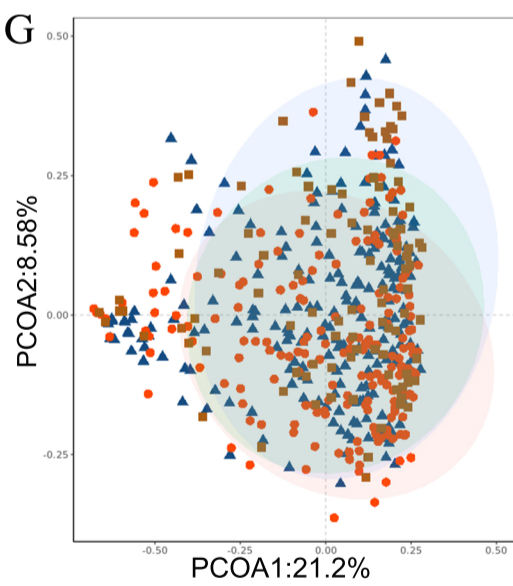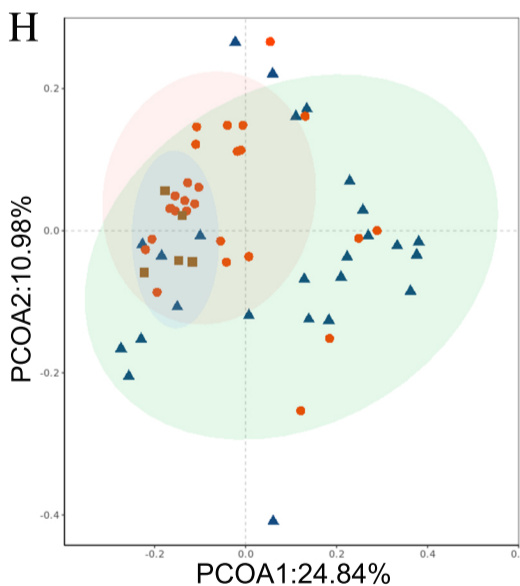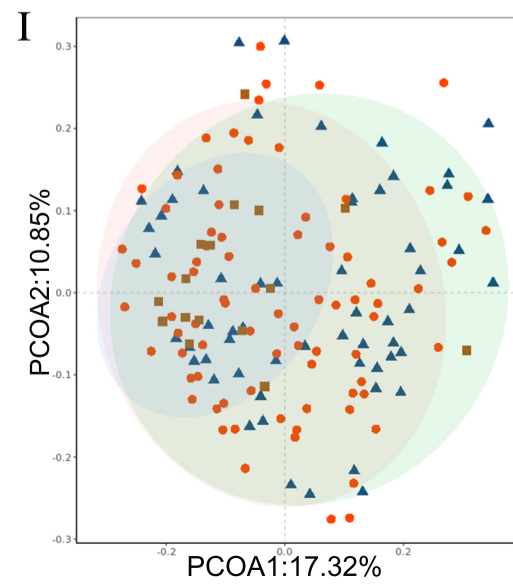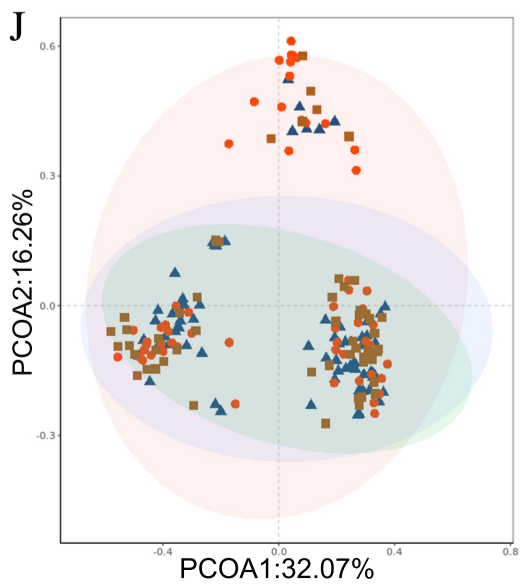

Supplement: Supplementary file 2 — Figure S2. [file CAM4-13-e70180-s006.pdf]

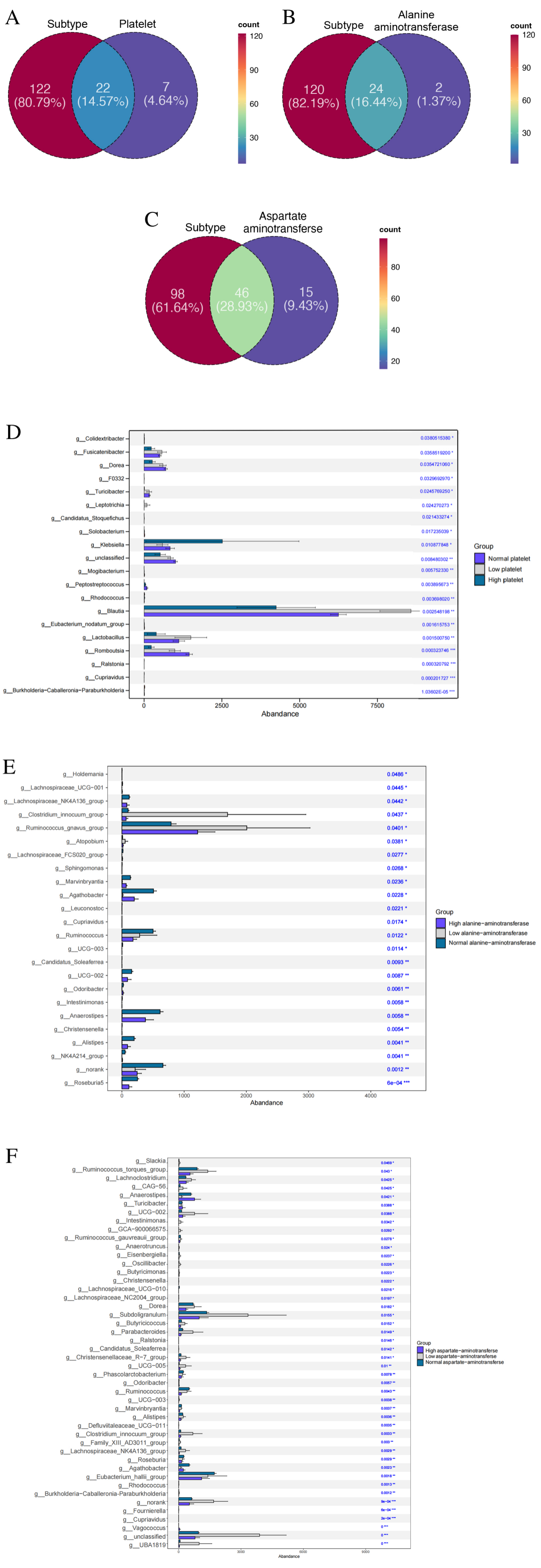

Supplement: Supplementary file 3 — Figure S3. [file CAM4-13-e70180-s003.pdf]

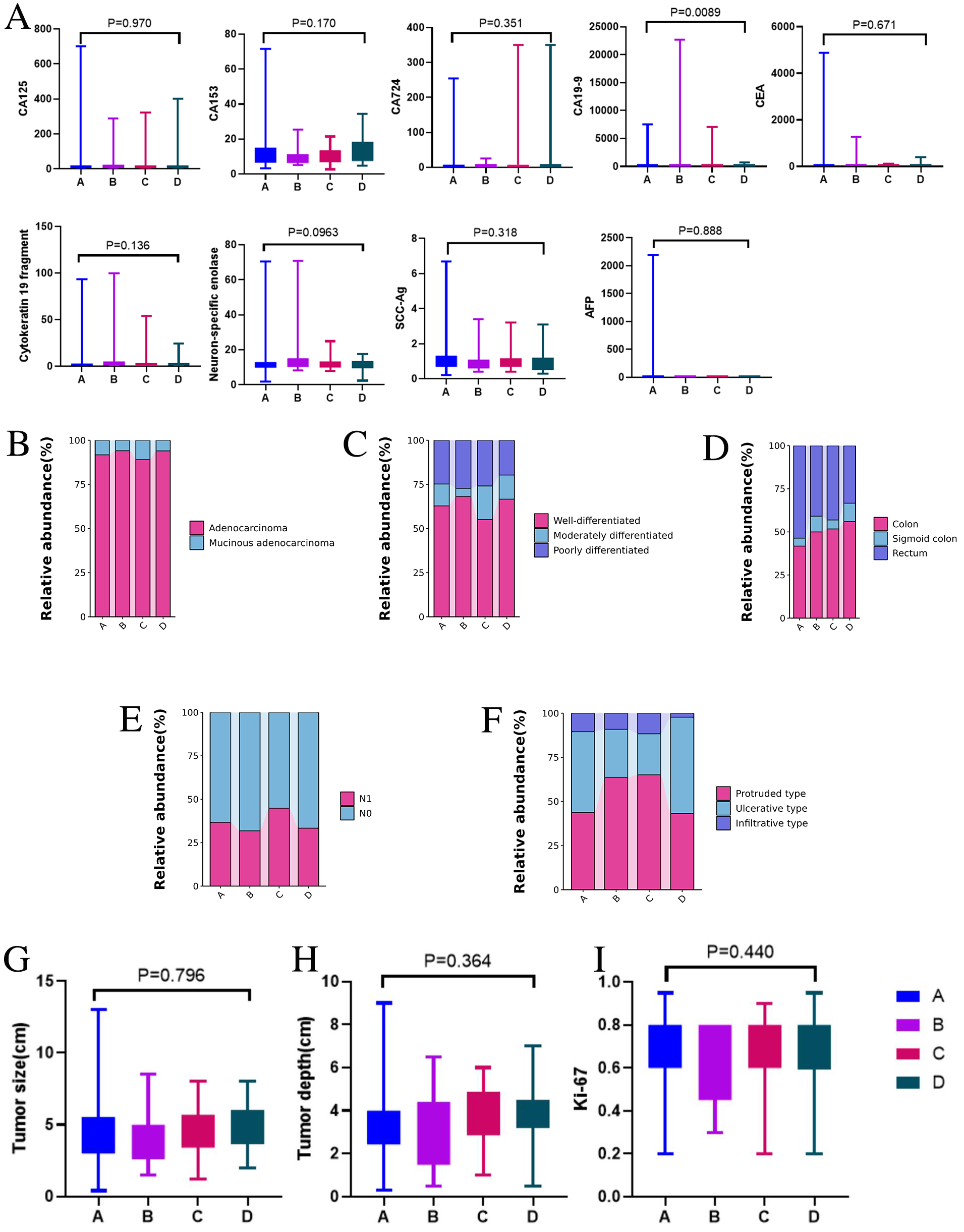

Supplement: Supplementary file 4 — Figure S4. [file CAM4-13-e70180-s007.tif]

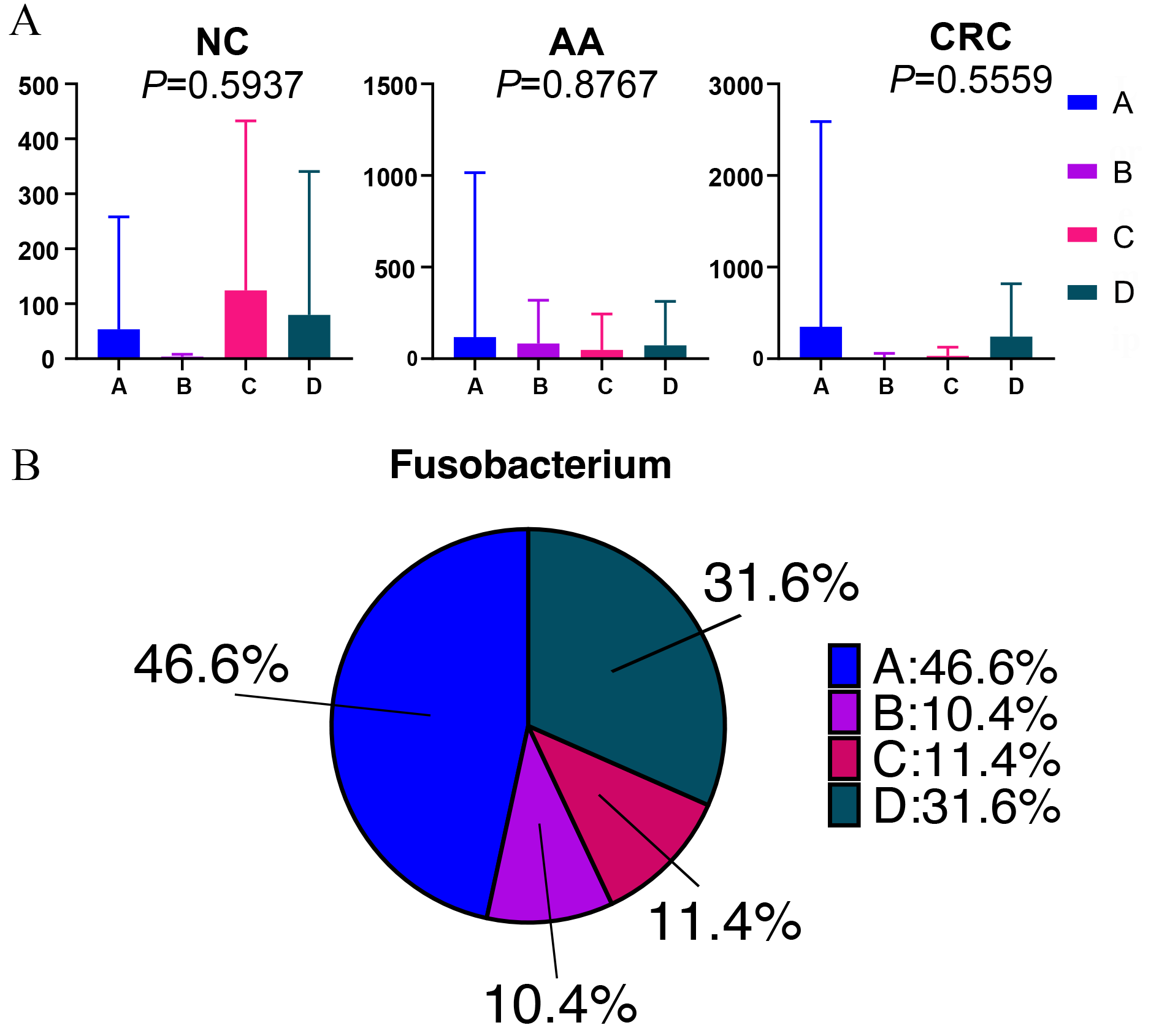

Supplement: Supplementary file 5 — Figure S5. [file CAM4-13-e70180-s004.tif]

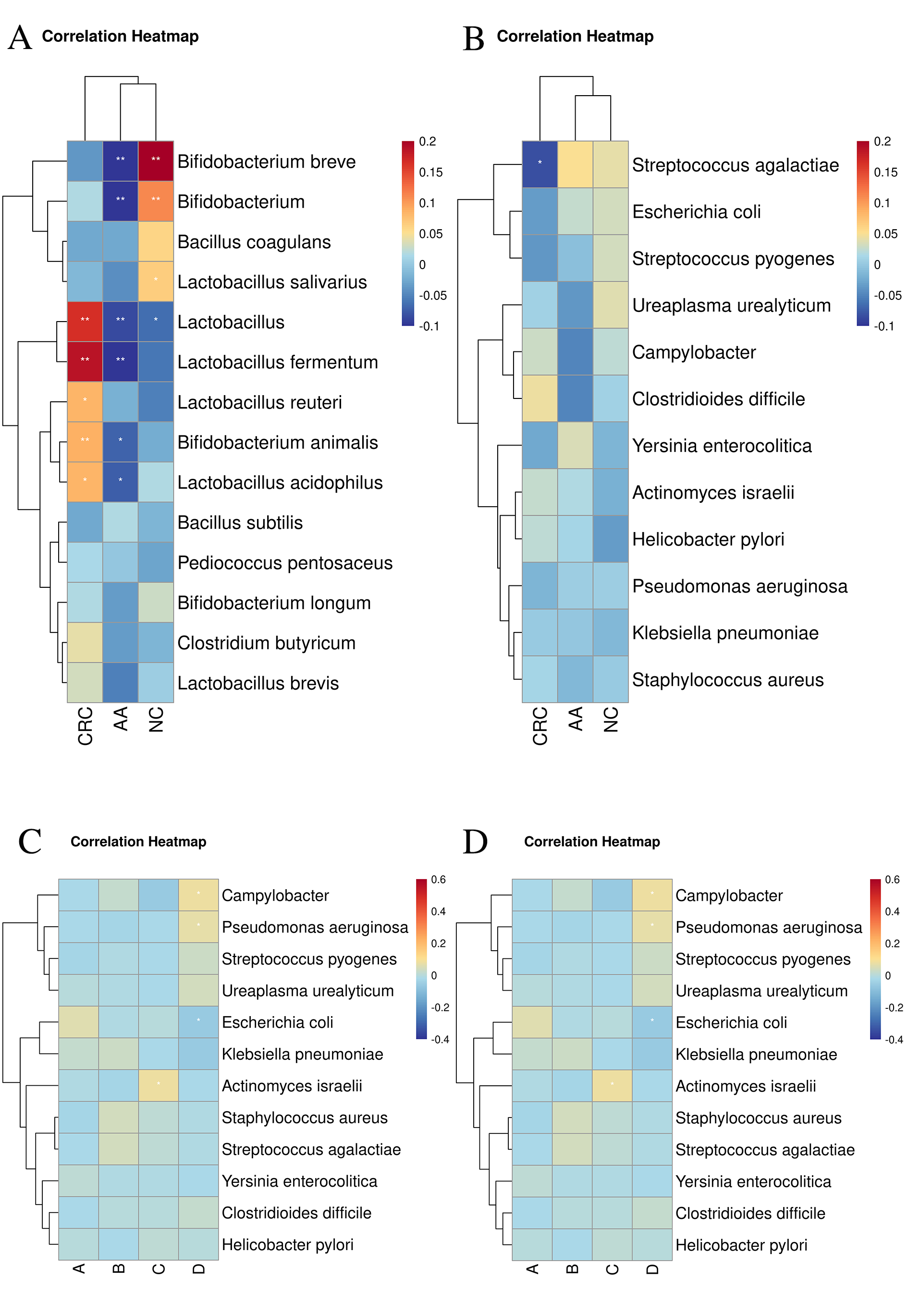

Supplement: Supplementary file 6 — Figure S6. [file CAM4-13-e70180-s002.tif]

A

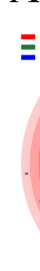

B

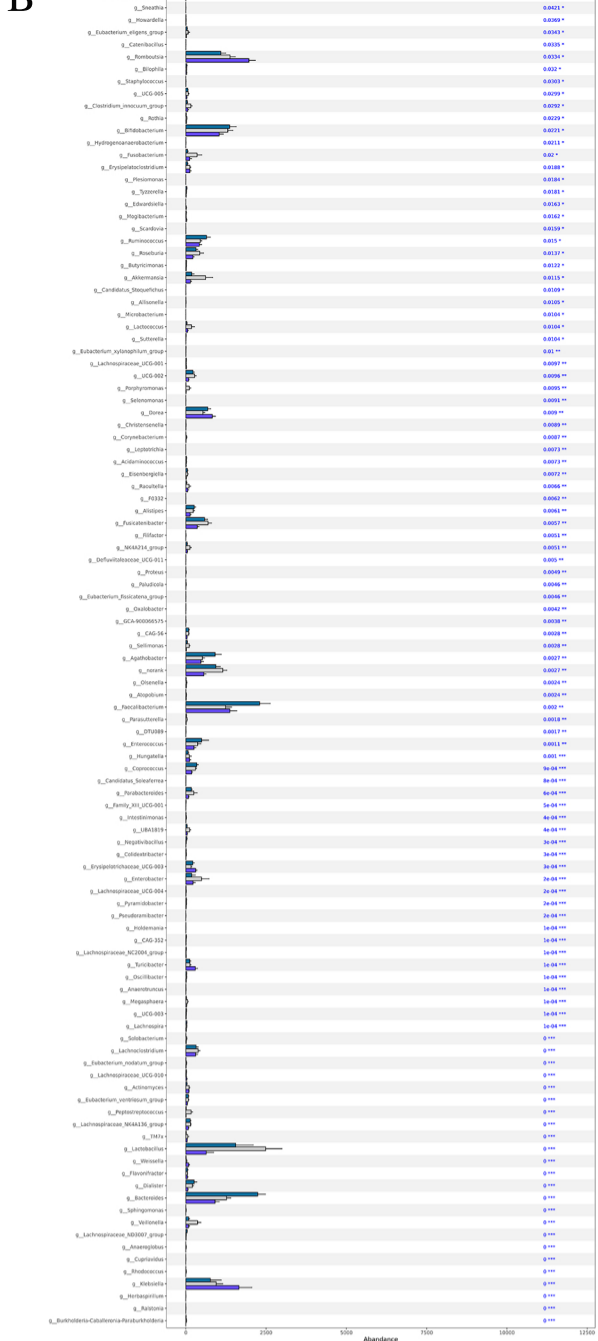

C

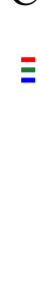

D

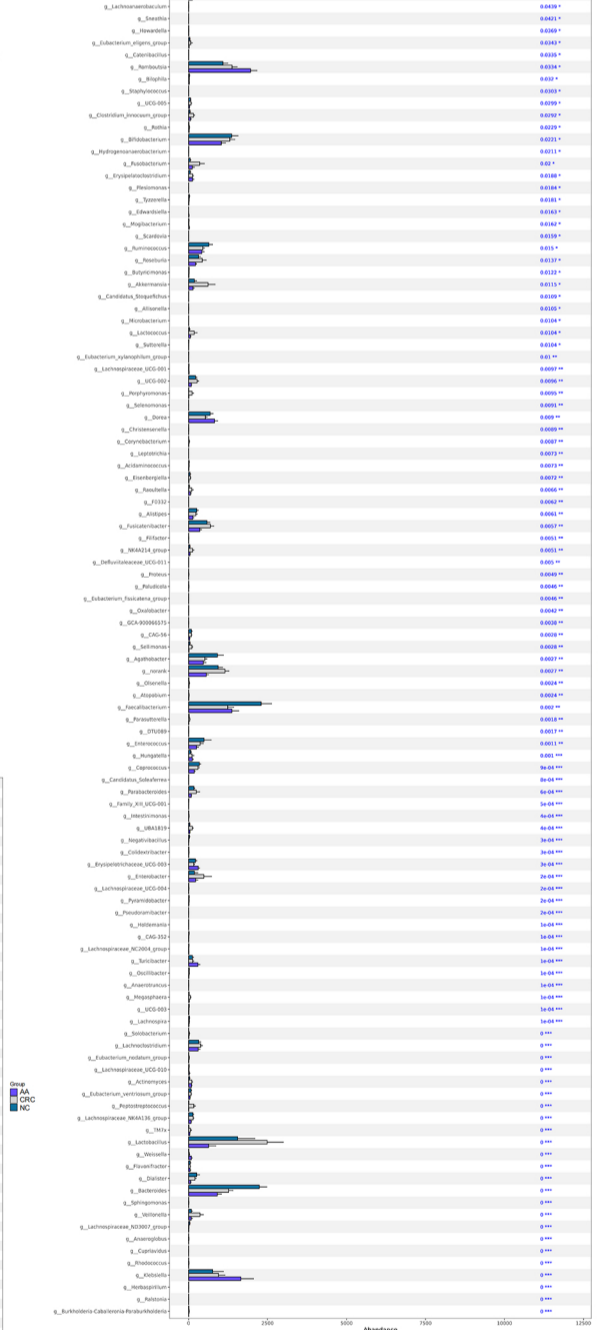

E

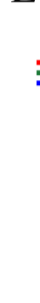

F

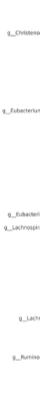

G

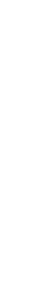

H

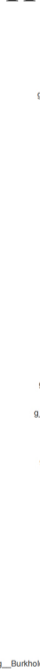

I

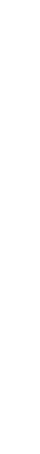

J

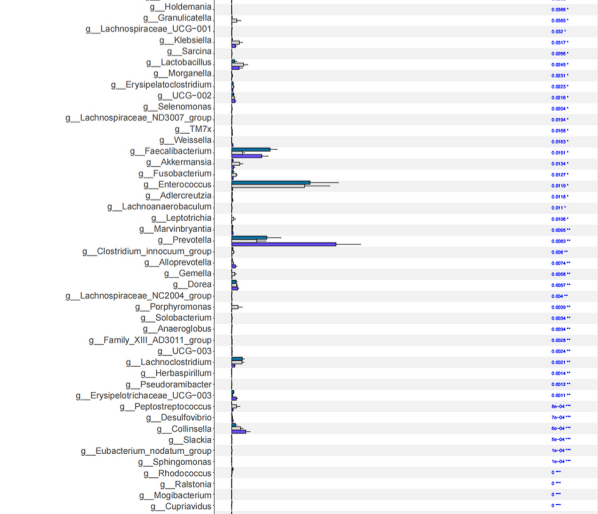

Supplement: Supplementary file 7 — Figure S7. [file CAM4-13-e70180-s001.pdf]

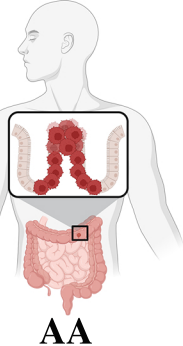

AA

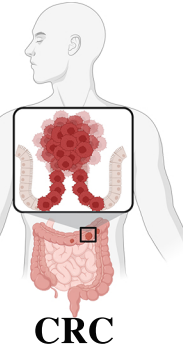

CRC

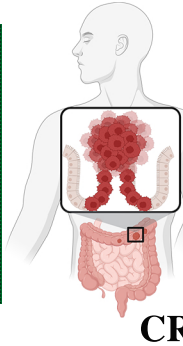

CRC/AA

## Colorectal disease

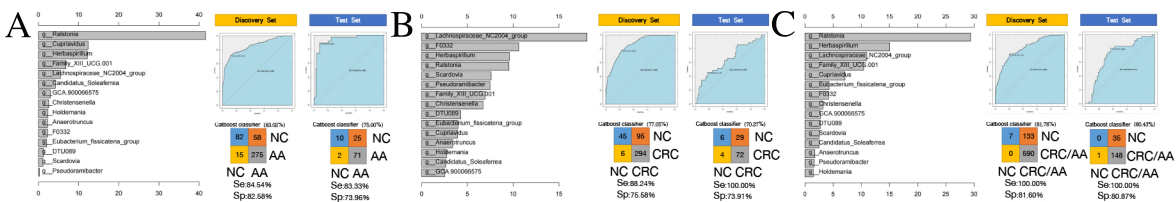

## Gut microbial subtype

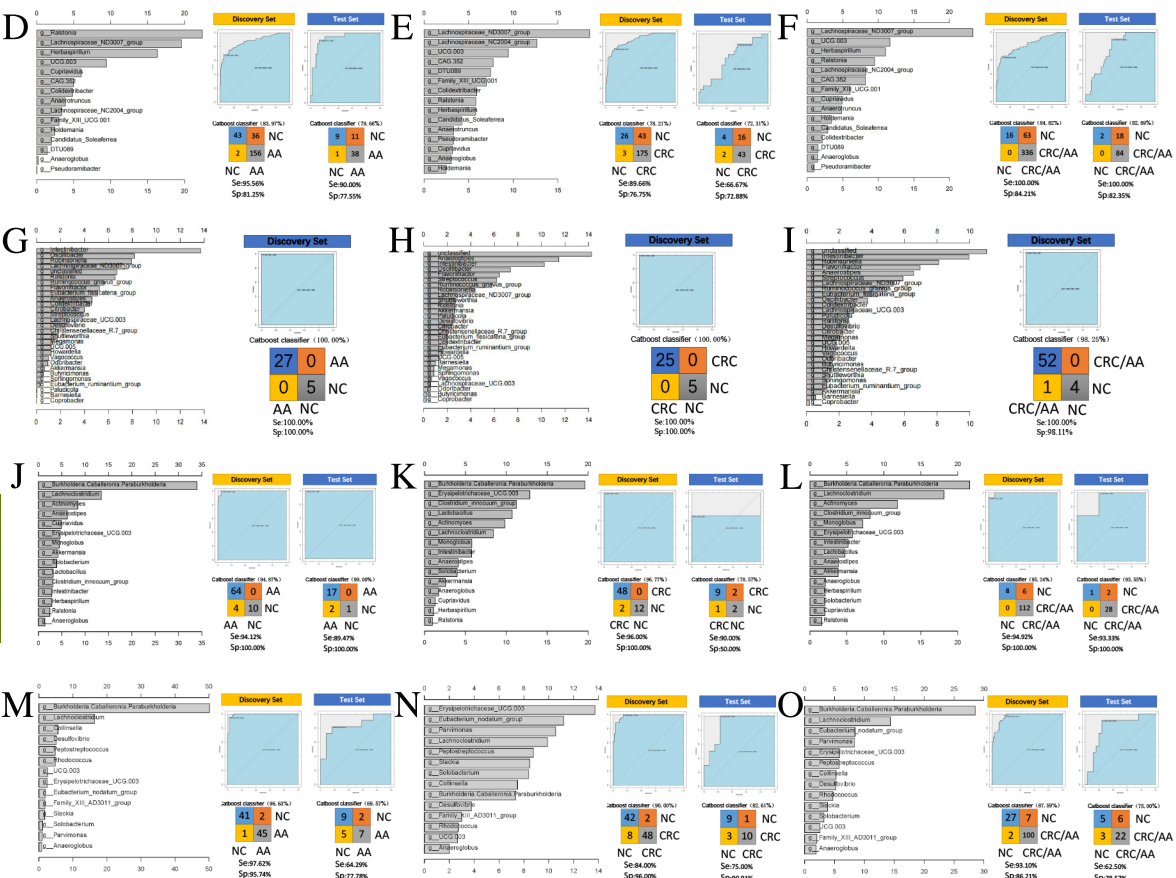

Supplement: Supplementary file 8 — Figure S8. [file CAM4-13-e70180-s005.pdf]
